# Supplementary material for: A homogeneous bioluminescent immunoassay to probe cellular signaling pathway regulation
Source: Commun Biol. 2020 Jan 3;3:8. doi: 10.1038/s42003-019-0723-9 (PMC6941952; doi:10.1038/s42003-019-0723-9)
Supplement: Supplementary file 1 — Supplementary Information [file 42003_2019_723_MOESM1_ESM.pdf]

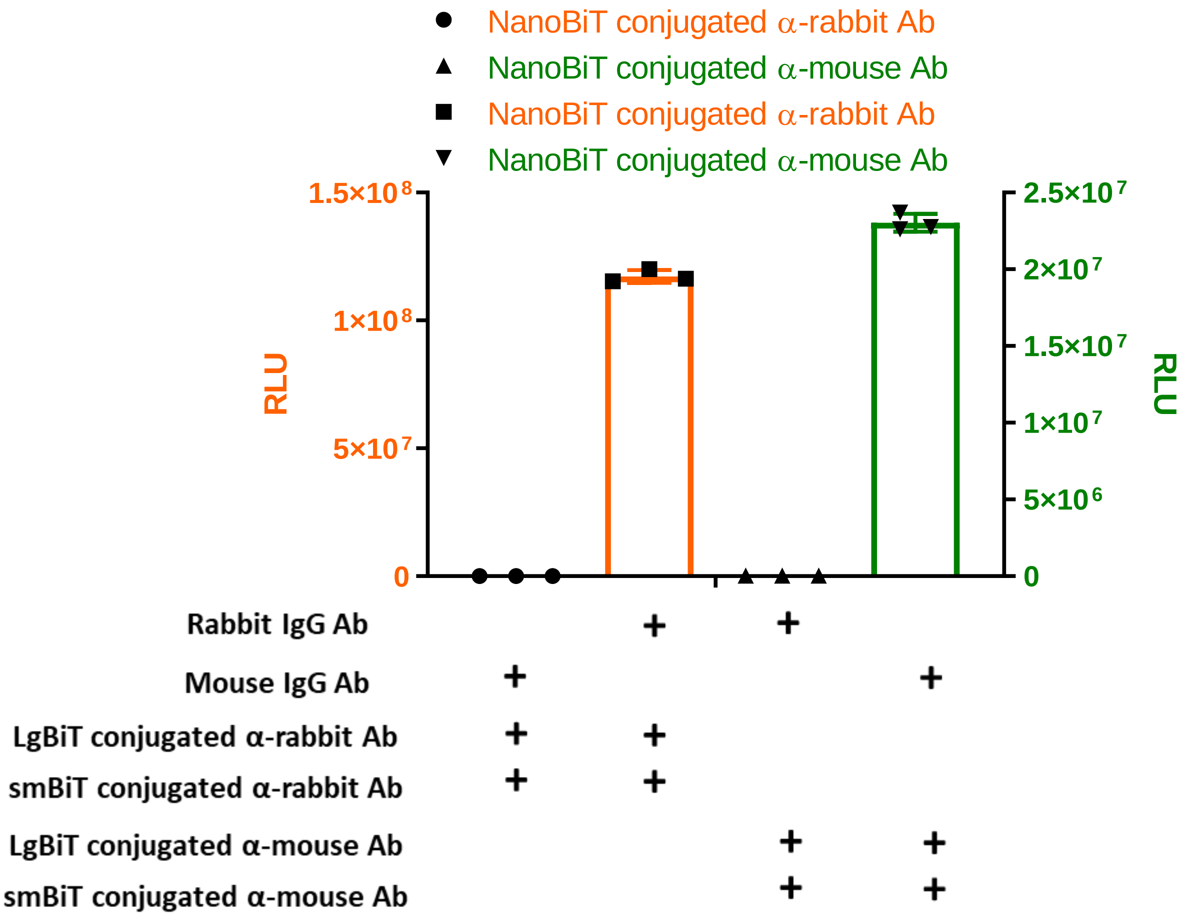

Supplementary Fig. 1: Specific binding of the NanoBiT-labeled secondary antibodies to their cognate IgG antibodies. Mouse or rabbit IgG antibodies were used as analytes and detected with either two NanoBiT-labelled anti-mouse or anti-rabbit secondary antibodies where one is labelled with Small BiT and the other labelled with the Large BiT fragments. Results are presented as means ± S.E.M. (n=3 technical replicates, the data are representative of two or more experiments).

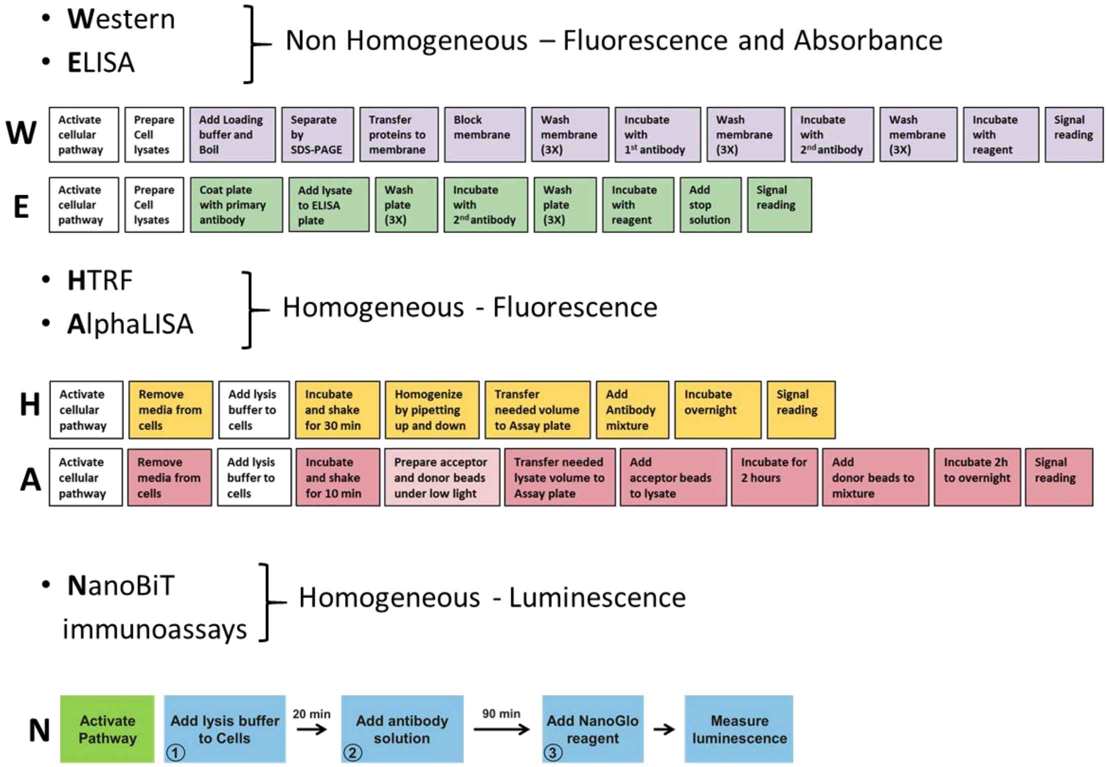

Supplementary Fig. 2: Comparison of the format of different immunoassay methods.

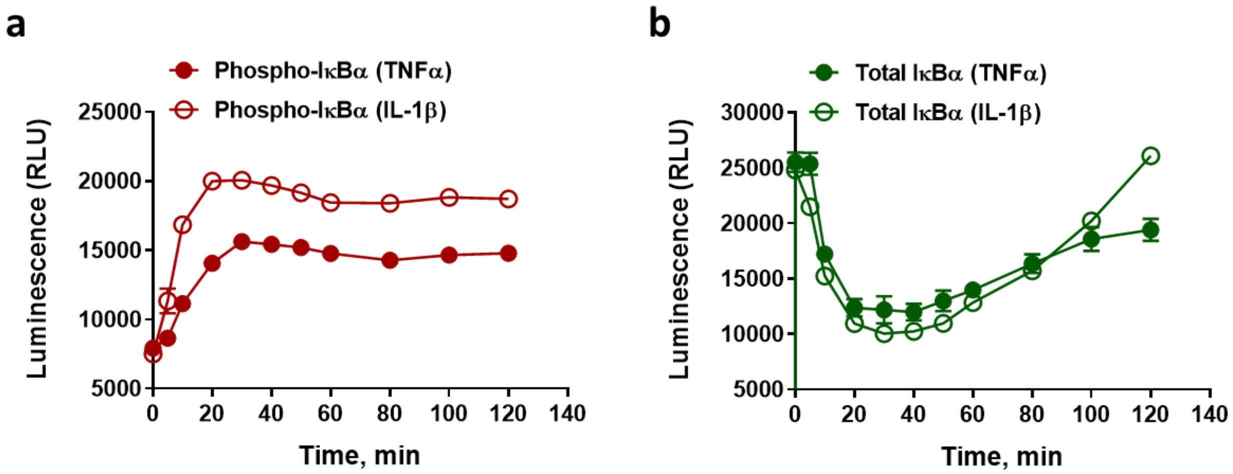

Supplementary Fig. 3: Detection of phosphorylated and total IκBα in NF-κB signaling pathway activated with two cytokines. 50,000 seeded MCF-7 cells were untreated or treated with TNFα (50ng/ml) or IL-1β (50ng/ml) for various time (5, 10, 20, 30, 40, 50, 60, 80, 100, and 120 min). a Phosphorylated or b total IκBα levels were measured by NanoBiT cell-based immunoassays.

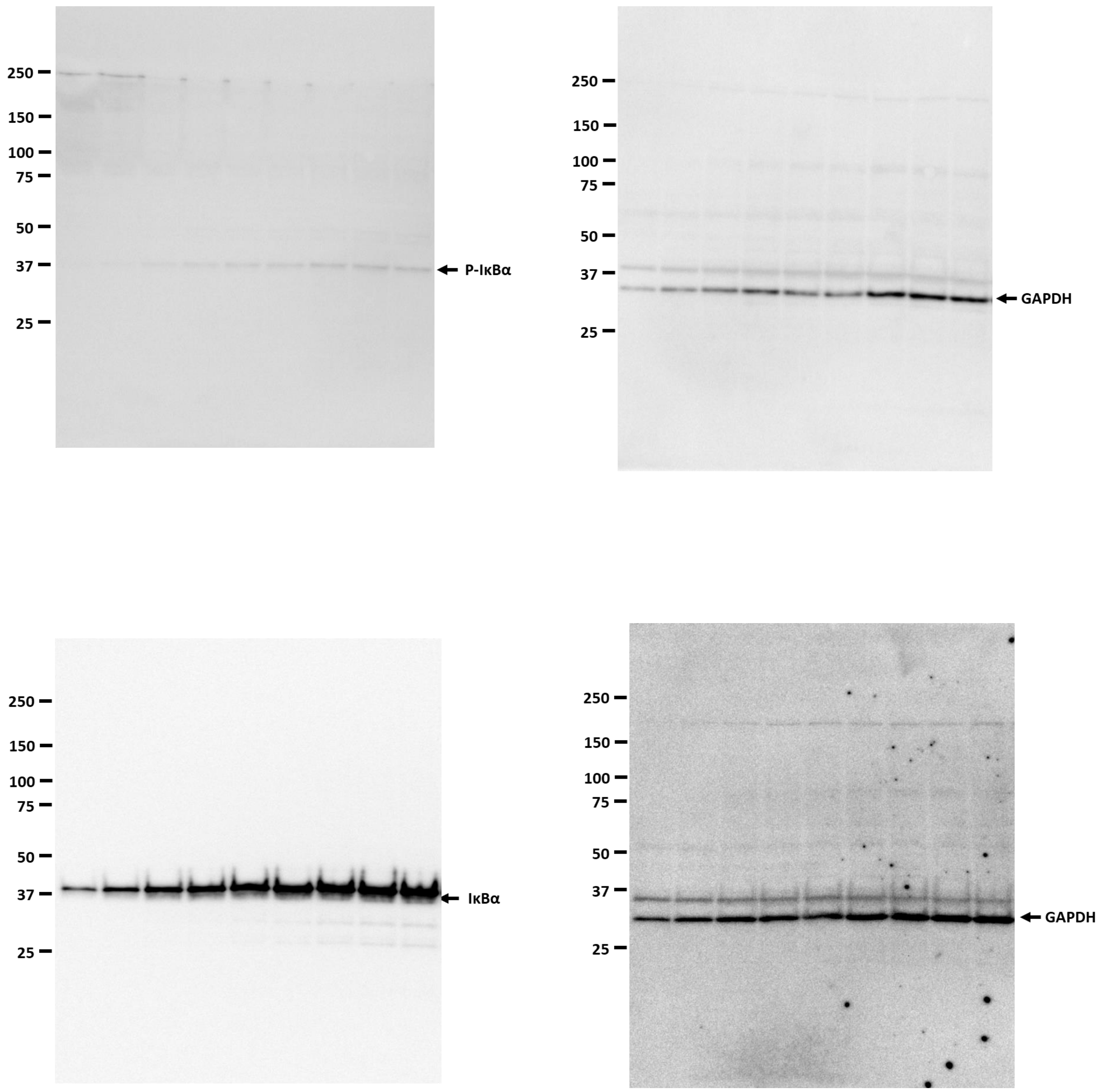

Supplementary Fig. 4. Original blot images for Fig. 3c and d
