# Supplementary material for: A homogeneous bioluminescent immunoassay to probe cellular signaling pathway regulation
Source: Commun Biol. 2020 Jan 3;3:8. doi: 10.1038/s42003-019-0723-9 (PMC6941952; doi:10.1038/s42003-019-0723-9)
Supplement: Supplementary file 2 — Description of additional supplementary files [file 42003_2019_723_MOESM2_ESM.pdf]

Description of additional supplementary items

SUPPLEMENTARY DATA 1 FILE LEGEND

Supplementary Data 1. Data for figures used in this study. Each tab corresponds to a given Figure.
