# Supplementary material for: A homogeneous bioluminescent immunoassay to probe cellular signaling pathway regulation
Source: Commun Biol. 2020 Jan 3;3:8. doi: 10.1038/s42003-019-0723-9 (PMC6941952; doi:10.1038/s42003-019-0723-9)
Supplement: Supplementary file 5 — Reporting Summary [file 42003_2019_723_MOESM5_ESM.pdf]

nature research

Corresponding author(s): Hicham Zegzouti

Last updated by author(s): Dec 3, 2019

Reporting Summary

Nature Research wishes to improve the reproducibility of the work that we publish. This form provides structure for consistency and transparency in reporting. For further information on Nature Research policies, see Authors & Referees and the Editorial Policy Checklist.

Statistics

For all statistical analyses, confirm that the following items are present in the figure legend, table legend, main text, or Methods section.

n/a	Confirmed	
☐	☒	The exact sample size (*n*) for each experimental group/condition, given as a discrete number and unit of measurement
☐	☒	A statement on whether measurements were taken from distinct samples or whether the same sample was measured repeatedly
☐	☒	The statistical test(s) used AND whether they are one- or two-sided<br>*Only common tests should be described solely by name; describe more complex techniques in the Methods section.*
☒	☐	A description of all covariates tested
☒	☐	A description of any assumptions or corrections, such as tests of normality and adjustment for multiple comparisons
☐	☒	A full description of the statistical parameters including central tendency (e.g. means) or other basic estimates (e.g. regression coefficient) AND variation (e.g. standard deviation) or associated estimates of uncertainty (e.g. confidence intervals)
☐	☒	For null hypothesis testing, the test statistic (e.g. *F*, *t*, *r*) with confidence intervals, effect sizes, degrees of freedom and *P* value noted<br>*Give P values as exact values whenever suitable.*
☒	☐	For Bayesian analysis, information on the choice of priors and Markov chain Monte Carlo settings
☒	☐	For hierarchical and complex designs, identification of the appropriate level for tests and full reporting of outcomes
☒	☐	Estimates of effect sizes (e.g. Cohen's *d*, Pearson's *r*), indicating how they were calculated

Our web collection on statistics for biologists contains articles on many of the points above.

Software and code

Policy information about availability of computer code

Data collection	GloMax® Discover 3.1.0., ImageQuant LAS 4000 version 1.1
Data analysis	GraphPad Prism8, Excel2016, ImageQuantTL 7.0

For manuscripts utilizing custom algorithms or software that are central to the research but not yet described in published literature, software must be made available to editors/reviewers. We strongly encourage code deposition in a community repository (e.g. GitHub). See the Nature Research guidelines for submitting code & software for further information.

Data

Policy information about availability of data

All manuscripts must include a data availability statement. This statement should provide the following information, where applicable:

- Accession codes, unique identifiers, or web links for publicly available datasets
- A list of figures that have associated raw data
- A description of any restrictions on data availability

The authors declare that the main data supporting the findings of this study are available within the article and its Supplementary Information files. Source data are available in Supplementary Data 1.

Field-specific reporting

Please select the one below that is the best fit for your research. If you are not sure, read the appropriate sections before making your selection.

☒ Life sciences ☐ Behavioural & social sciences ☐ Ecological, evolutionary & environmental sciences

Life sciences study design

All studies must disclose on these points even when the disclosure is negative.

Sample size	Sample size was indicated in the manuscript.
Data exclusions	No data was excluded from the analyses.
Replication	All attempts at replication were successful.
Randomization	Not relevant for this study. Not clinical study
Blinding	Not relevant for this study. Not clinical study

Reporting for specific materials, systems and methods

We require information from authors about some types of materials, experimental systems and methods used in many studies. Here, indicate whether each material, system or method listed is relevant to your study. If you are not sure if a list item applies to your research, read the appropriate section before selecting a response.

Materials & experimental systems

n/a	Involved in the study	
☐	☒	Antibodies
☐	☒	Eukaryotic cell lines
☒	☐	Palaeontology
☒	☐	Animals and other organisms
☒	☐	Human research participants
☒	☐	Clinical data

Methods

n/a	Involved in the study	
☒	☐	ChIP-seq
☒	☐	Flow cytometry
☒	☐	MRI-based neuroimaging

Antibodies

Antibodies used	These antibodies were used at 150ng/ml. Cell signaling Technology Inc.: Rabbit anti-phospho-IκBα (#2859), Mouse anti-phospho-IκBα (#9246), Mouse anti-IκBα (#4814), Mouse anti-IκBα (#9247), Rabbit anti-IκBα (#4812), Mouse anti-phospho-p65 (#13346), Rabbit anti-p65 (#8242), Mouse anti-p65 (#6956), Mouse anti-phospho-AKT (#4051), Rabbit anti-AKT (#4691), Mouse anti-AKT (#2966), Rabbit anti-phospho-STAT3 (#9145), Mouse anti-STAT3 (#9139), and Rabbit anti-STAT3 (#8768). ThermoScientific: Rabbit anti-ER (#MA5-14501) and Mouse anti-ER (#MA5-13191). This antibody was used in a titration from 0.0012-80 µg/ml. Genentech: ACTEMRA® (tocilizumab, # NDC 50242-136-01). This antibody was used in a titration from 0.76ng/ml - 20 µg/ml. R&D System: Anti-human IL-6 antibody (#AF-206-NA).
Validation	Antibody validation can be found in each manufacture company's website.

Eukaryotic cell lines

Policy information about cell lines

Cell line source(s)	MCF-7, Hela, Ramos-RA1, A431, HEK293, THP-1 cells were purchased from American Type Culture Collection (ATCC). Human Primary Mammary Epithelial Cells and Human Breast Tumor Epithelial Cells were purchased from Cell Biologics.
Authentication	Authentication must be performed in supplying companies. We didn't do authentication by ourselves.
Mycoplasma contamination	Mycoplasma contamination of the cell lines was not tested.
Commonly misidentified lines (See ICLAC register)	No misidentified lines were used in this study.
